# Friction in Insomnia

**Published:** 1880-08

**Authors:** F. Peterson


### FRICTION IN INSOMNIA.

FROM THE FRENCH BY F. PETERSON, M. D.

Nervous persons are more than all others subject to sleeplessness. To obtain a little sleep they have recourse to narcotics which always end by having a pernicious influence on the health. We can recommend to such a very simple method, and which infallibly procures the repose many seek by other means: this is the rubbing of the body, or friction for some minutes before retiring, either with a piece of coarse woolen cloth, or, if preferred, with a friction-brush.—*La Lancette Belge.*

